# *QuickStats:* Percentage* of Current Cigarette Smokers^†^ Aged ≥18 Years Who Received Advice from a Health Professional To Quit Smoking,^§^ by Sex and Age Group — United States, 2022

**DOI:** 10.15585/mmwr.mm7324a5

**Published:** 2024-06-20

**Figure:**
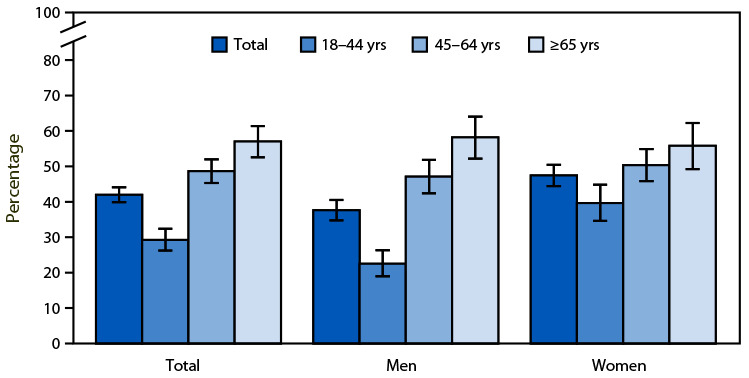
In 2022, 42.0% of current cigarette smokers aged ≥18 years received advice from a doctor, dentist, or other health professional about ways to quit smoking. The percentage of current smokers who received advice to quit smoking increased with age. Overall, and for current smokers aged 18–44 years, men were less likely to receive advice on quitting compared with women.

**Supplementary Table:** https://stacks.cdc.gov/view/cdc/156763

**Source:** National Center for Health Statistics, National Health Interview Survey, 2022. https://www.cdc.gov/nchs/nhis.htm

For more information on this topic, CDC recommends the following link: https://www.cdc.gov/tobacco/index.html

